# Two-hour dynamic reassessment of noninvasive ventilation failure risk in acute exacerbation of chronic obstructive pulmonary disease with hypercapnic respiratory failure: development and temporal validation

**DOI:** 10.3389/fmed.2026.1920677

**Published:** 2026-08-14

**Authors:** Tao Liu, Xiaohao Yan, Jianchao Lu

**Affiliations:** 1 Solid-State Fermentation Resource Utilization Key Laboratory of Sichuan Province, Faculty of Agriculture, Forestry and Food Engineering, Yibin University, Yibin, Sichuan, China; 2 Chengdu Huake Biology Research Center, Chengdu, Sichuan, China; 3 Department of Radiation Oncology, Radiation Oncology Key Laboratory of Sichuan Province, Sichuan Clinical Research Center for Cancer, Sichuan Cancer Hospital and Institute, Sichuan Cancer Center, Affiliated Cancer Hospital of University of Electronic Science and Technology of China, Chengdu, Sichuan, China

**Keywords:** acute exacerbation of chronic obstructive pulmonary disease, dynamic reassessment, HACOR, hypercapnic respiratory failure, NIV failure, noninvasive ventilation, risk stratification, temporal validation

## Abstract

**Background:**

Early recognition of noninvasive ventilation (NIV) failure remains difficult in acute exacerbation of chronic obstructive pulmonary disease (AECOPD) with hypercapnic respiratory failure. We developed and temporally validated a model for structured reassessment after early NIV treatment and compared it with a pre-NIV model and the heart rate, acidosis, consciousness, oxygenation, and respiratory rate (HACOR) score.

**Methods:**

The hospital information system identified consecutive NIV-treated AECOPD admissions from 2021 through 2025. After prespecified data-consistency exclusions, 924 first eligible hospitalizations were divided by calendar time into development and temporal-validation cohorts. The dynamic model used the first complete physiological assessment obtained 90–150 min after NIV initiation. Discrimination, calibration, clinical utility, and robustness were evaluated; detailed analytic methods and supporting results are provided in the Supplementary material.

**Results:**

NIV failure occurred in 136 of 692 patients in development and 51 of 232 in temporal validation. The dynamic model retained good discrimination and close calibration in validation. Its numerical performance was slightly better than the pre-NIV model and HACOR-derived probability, but superiority was not established. Respiratory rate and level of consciousness at reassessment carried the clearest independent prognostic information, whereas the contributions of acidosis, oxygenation, and pneumonia were more context dependent.

**Conclusion:**

The model provides a reproducible probability estimate for structured early treatment-response reassessment, but it does not replace HACOR, clinical judgment, or established escalation criteria. External validation and prospective impact evaluation are required.

## 1 Introduction

Chronic obstructive pulmonary disease (COPD) remains a major cause of morbidity, mortality, and healthcare use worldwide, and acute exacerbations account for much of its short-term clinical burden. Acute or acute-on-chronic hypercapnic respiratory failure represents one of the most severe presentations. In appropriately selected patients, noninvasive ventilation (NIV) unloads the respiratory muscles, improves alveolar ventilation, and reduces endotracheal intubation and in-hospital mortality. These benefits have established bilevel NIV as first-line ventilatory support for acidotic COPD exacerbations when no immediate indication for invasive ventilation is present (1–8).

The clinical challenge does not end when NIV is started. Some patients improve rapidly, whereas others remain exposed to respiratory muscle fatigue, secretion retention, worsening gas exchange, impaired consciousness, hemodynamic instability, or progression of the precipitating illness. Failure may occur during the initial hours or later in the admission, and delayed recognition can prolong ineffective support and defer necessary escalation. Guidelines therefore emphasize close physiological observation, repeated clinical assessment, and timely access to invasive ventilation rather than an unstructured trial continued solely because NIV has already been initiated (9–13).

Early treatment response is clinically informative because NIV functions as a brief physiological challenge. A fall in respiratory rate suggests reduced ventilatory load and more effective unloading; improvement in consciousness may indicate correction of hypercapnic encephalopathy; and changes in pH, carbon dioxide, and oxygenation reflect the combined effects of ventilation, oxygen delivery, pulmonary pathology, and treatment tolerance. Previous studies incorporated measurements obtained after treatment began and repeatedly identified persistent tachypnea, impaired consciousness, and limited correction of gas exchange as markers of an unfavorable trajectory (14–16). The reassessment window therefore addresses a different question from baseline severity: not only how ill the patient was at presentation, but whether the initial NIV strategy is producing the expected physiological response.

The heart rate, acidosis, consciousness, oxygenation, and respiratory rate (HACOR) score translates several of these domains into a rapid bedside instrument. Its simplicity is a major advantage, although transport across settings may alter operating characteristics and a point score does not directly provide a calibrated probability for action-specific decisions. The Noninvasive Ventilation Outcomes score addresses another clinically relevant but distinct target—hospital mortality among patients requiring assisted ventilation—rather than failure of an ongoing NIV trial. Mortality prediction, intubation risk, and the need for intensified reassessment overlap, but they are not interchangeable purposes (17–19).

Prediction research in COPD has also been limited by heterogeneous outcome definitions, modest event counts, incomplete model presentation, and insufficient attention to calibration and validation. A model can rank patients reasonably well while producing probabilities that are too extreme or too moderate, and numerical performance does not by itself show that a tool improves care. Contemporary reporting and appraisal frameworks consequently emphasize clear timing of predictor measurement, transparent handling of missing or inconsistent data, complete model specification, temporal or external validation, calibration, and a clinically explicit intended use (20–25).

A further challenge is translating model output into a clinical action. Discrimination describes relative ranking, but clinicians also need to know whether predicted probabilities agree with observed risk and whether a proposed threshold is appropriate for the intended response. A probability used to trigger closer observation can tolerate a different balance of false-positive and false-negative classifications from one linked to invasive ventilation. Calibration, decision-curve analysis, and explicit definition of the intended use are therefore necessary complements to the area under the curve, but none can replace prospective evaluation of a specified care pathway (26–28).

We therefore developed a pre-NIV model based on information available at treatment initiation and a dynamic model incorporating the first complete physiological reassessment after NIV began. Both models were evaluated in a later calendar-year cohort and compared with HACOR. The objective was to determine whether early treatment response could provide a reproducible probability estimate for structured reassessment while preserving a conservative clinical interpretation: the model was not intended to predict before treatment response, replace HACOR or bedside judgment, or supersede conventional criteria for immediate escalation.

## 2 Materials and methods

### 2.1 Study design and setting

This retrospective cohort study used hospital information-system and electronic-record data from consecutive adults treated with NIV for AECOPD and hypercapnic respiratory failure at Sichuan Cancer Hospital & Institute. Only the first hospitalization meeting all eligibility criteria was retained for each patient. Admissions from 2021 to 2024 formed the development cohort and admissions from 2025 formed a temporally separate validation cohort. The study followed TRIPOD and TRIPOD+AI recommendations, and a completed checklist with section and page mapping is supplied separately (23, 25).

The temporal-validation data were held apart from model development. They were not used for predictor selection, coefficient estimation, cutoff selection, or construction of the HACOR probability transformation. This calendar-time design was chosen to test transport across a later period within the same hospital rather than random resampling of contemporaneous records.

### 2.2 Participants and record-level eligibility

Eligibility was based on the treating respiratory or critical-care team’s diagnosis of AECOPD with hypercapnic respiratory failure and documented NIV treatment. The intended-use population comprised patients who remained alive, had not undergone intubation, and had not otherwise met immediate escalation criteria by the prespecified reassessment window. Patients who deteriorated before that landmark were outside the model’s target population.

Prespecified endpoint and length-of-stay consistency rules were applied before analysis. The coded NIV-failure status had to agree with the component event fields and recorded time to failure, and ICU length of stay could not exceed total hospital stay. Records with unresolved discrepancies were excluded without imputation or retrospective recoding. Full consistency rules, overlap among exclusions, and comparisons between included and excluded records are reported in Supplementary Table S8.

### 2.3 Clinical management and data collection

NIV management followed routine clinical care rather than a study-specific protocol. Candidate predictors were drawn from information available before NIV and from the first complete physiological assessment obtained 90–150 min after documented delivery of positive-pressure ventilation. When several eligible assessments were available, the assessment closest to 120 min was selected. Bedside variables and arterial blood-gas measurements were required to originate from the same assessment episode.

The pre-NIV variables represented recent exacerbation burden, home oxygen use, congestive heart failure, pneumonia-triggered exacerbation, treatment timing, vital signs, gas exchange, and laboratory markers. The dynamic update added respiratory rate, Glasgow Coma Scale (GCS), arterial pH, and oxygenation at reassessment. Oxygen records were retrospectively harmonized with blood-gas sampling; FiO₂ provenance, stability rules, and exclusion of an unreconciled pre-NIV oxygenation field are described in Supplementary material.

### 2.4 Outcome definition

The primary outcome was a documented composite of endotracheal intubation, ICU transfer, or in-hospital death after NIV initiation. The components were clinically important but not equivalent. In particular, ICU transfer could reflect closer monitoring or organizational practice rather than definitive physiological failure of NIV. Hard-endpoint, intubation-only, early-failure, and component-pattern analyses were therefore prespecified, and the detailed outcome rules are provided in Supplementary material.

### 2.5 Candidate predictors and model specification

Predictors were selected before model fitting from bedside availability and prior clinical evidence rather than univariable statistical significance. The pre-NIV model contained the baseline clinical and physiological predictors, whereas the dynamic model added the four reassessment variables. This preserved the clinical order in which information becomes available and allowed the added contribution of early treatment response to be examined. The complete predictor list and coefficients are provided in Supplementary Table S2.

Continuous predictors were retained on their measured scales, with clinically interpretable rescaling of pH and oxygenation. No data-driven categorization or stepwise selection was used for the primary model. Logistic regression was fitted in the development cohort, and the resulting intercept and coefficients were applied unchanged in temporal validation. Functional-form assessment, the complete equation, predictor coding, and the offline calculator are reported in the Supplementary material.

### 2.6 HACOR comparator

HACOR was calculated at reassessment according to the published scoring system (17). The raw integer score was used for discrimination and classification at its conventional bedside threshold. Because calibration and decision-curve analysis require a probability, a logistic transformation of raw HACOR was fitted in development and applied unchanged in temporal validation. The transformation and its validation performance are described in Supplementary material and Table 1.

**Table 1:** Temporal-validation performance of the pre-NIV model, dynamic model, and HACOR-derived probability.

Model	AUC (95% CI)	Calibration intercept (95% CI)	Calibration slope (95% CI)	Brier score
Pre-NIV model	0.758 (0.683–0.832)	0.165	1.280	0.142
Dynamic model	0.804 (0.741–0.866)	0.041	0.939	0.137
HACOR-derived probability	0.781 (0.715 to 0.847)	0.015 (−0.337 to 0.367)	0.879 (0.573 to 1.185)	0.145

AUC, calibration, and Brier score are shown for the temporal-validation cohort to emphasize out-of-sample performance. The HACOR-derived probability was obtained by logistic recalibration in the development cohort and applied unchanged in validation; its AUC is identical to that of the raw HACOR score. Detailed development performance, classification, and threshold analyses are provided in the Supplementary material. CI, confidence interval; HACOR, heart rate, acidosis, consciousness, oxygenation, and respiratory rate; NIV, noninvasive ventilation.

### 2.7 Model performance and classification

Model performance was evaluated by discrimination, calibration, overall prediction error, and graphical uncertainty. Calibration plots combined a flexible curve with bootstrap uncertainty and grouped observed outcome proportions. Classification at development-derived and fixed probability thresholds was explicitly treated as exploratory. The main text emphasizes temporal-validation performance, while development estimates, threshold tables, and confidence-interval methods are reported in the Supplementary material (26).

### 2.8 Decision-curve and clinical impact analyses

Decision-curve and clinical-impact analyses assessed the potential consequences of risk-guided reassessment across clinically plausible threshold probabilities. These analyses were interpreted as support for intensified observation, repeat testing, or senior review, not as evidence for an automatic intubation rule (27, 28).

### 2.9 Sensitivity and robustness analyses

Robustness analyses evaluated harder outcomes, intubation alone, early failure, exclusion of pneumonia-triggered exacerbations, functional form, and selection associated with excluded records. Ridge and elastic-net regression assessed shrinkage, and exploratory reduced models tested whether the reassessment variables alone provided adequate performance. Internal optimism was estimated by bootstrap resampling. Full model specifications, penalty selection, reduced-model comparisons, and weighted analyses are reported in Supplementary Tables S3–S14 (29).

### 2.10 Sample size, missing data, and statistical analysis

The retrospective cohort included all eligible patients and was not defined by a prospective sample-size calculation. A *post hoc* development-sample-size assessment used the Riley framework and is reported as a descriptive check rather than an *a priori* justification. The temporal-validation cohort was fixed by the available later-period admissions, and its event count was recognized as limiting the precision of calibration and model comparisons (30, 31).

Primary-model and comparator variables were complete, so no imputation was performed. Continuous variables were summarized using means with standard deviations or medians with interquartile ranges, and categorical variables as counts and percentages. Between-group comparisons used parametric or rank-based tests for continuous variables and chi-square or Fisher exact tests for categorical variables, as appropriate.

Analyses were performed in R version 4.5.3. Tests were two-sided, and interpretation emphasized effect estimates, confidence intervals, calibration, and clinical meaning rather than isolated significance thresholds. The analysis code and model specification are available under the data-availability conditions.

### 2.11 Ethics statement

The study protocol was reviewed and approved by the Ethics Committee of Sichuan Cancer Hospital & Institute (approval no. scch2511032). The committee waived the requirement for written informed consent because the study used retrospectively collected, de-identified clinical data. The study was conducted in accordance with the Declaration of Helsinki and applicable institutional requirements. The study was not prospectively registered. Patients and members of the public were not involved in the design, conduct, reporting, or dissemination planning of this retrospective study.

## 3 Results

### 3.1 Cohort assembly and temporal split

The source cohort contained 1,040 first eligible hospitalizations. Prespecified consistency checks excluded 116 records, leaving 924 patients for analysis (z). The development cohort included 692 patients and the temporal-validation cohort 232 patients; NIV failure occurred in 136 and 51 patients, respectively. All retained patients reached the reassessment window without earlier intubation or another immediate escalation event.

**Figure 1:**
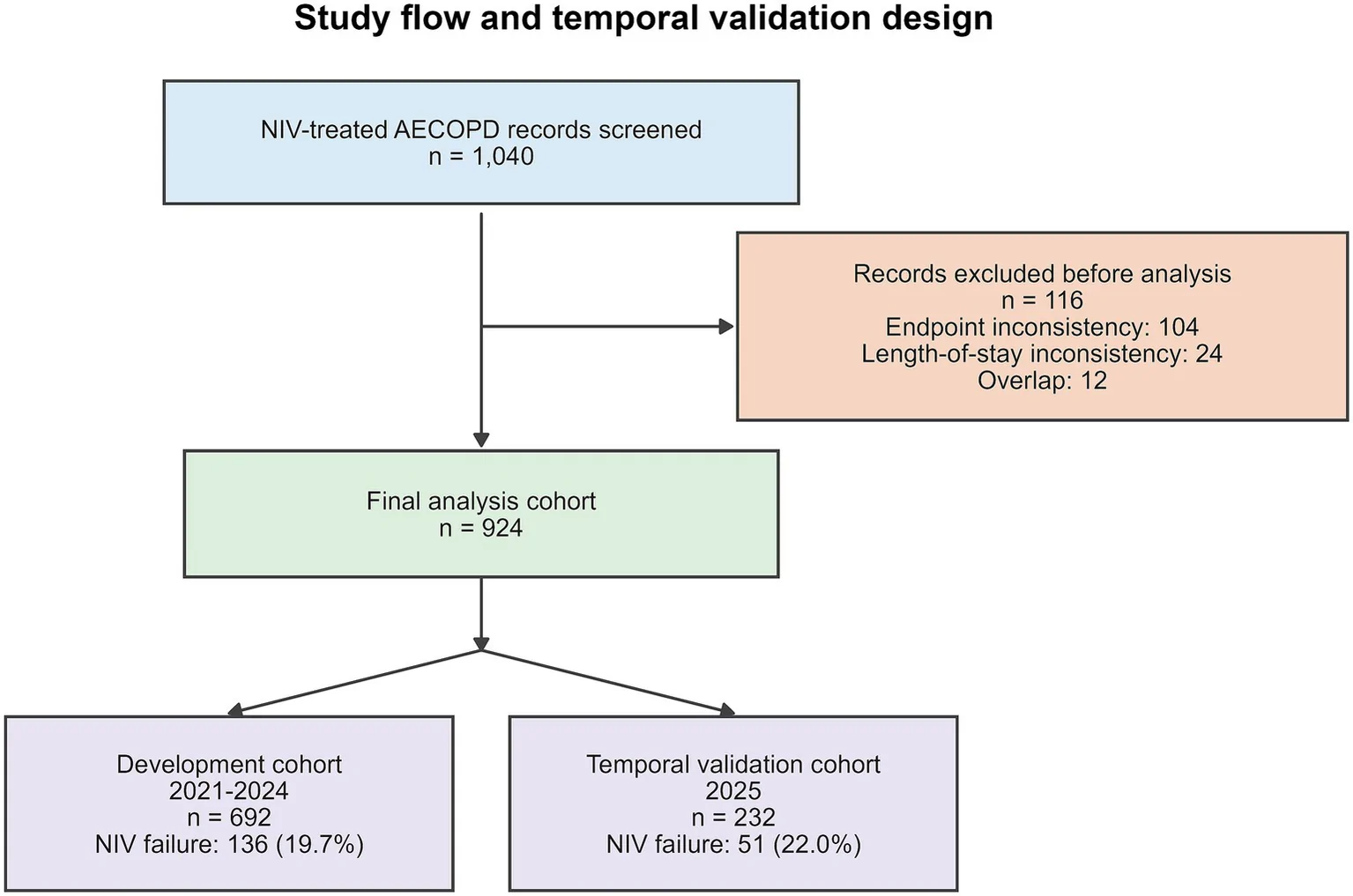
Study flow and temporal cohort allocation. The hospital information system identified 1,040 records, with only the first eligible hospitalization retained per patient. After exclusion of 116 unique records with unresolved endpoint inconsistency or impossible length-of-stay values, 924 patients remained: 692 in the 2021–2024 development cohort and 232 in the 2025 temporal validation cohort.

Development and validation cohorts were broadly comparable, with the detailed distributions shown in Supplementary Table S1. Included and excluded records were also generally similar, although the excluded group had evidence of slightly greater baseline acidosis. Inverse-probability-of-inclusion weighting produced results close to the primary analysis, while appropriately leaving open the possibility of selection related to unmeasured factors (Supplementary Tables S8, S12).

### 3.2 Baseline and pre-NIV characteristics by outcome

Patients with NIV failure more often had home oxygen use and pneumonia-triggered exacerbation and presented with faster heart and respiratory rates, greater acidosis, and higher carbon dioxide levels. Age, sex, body mass index, prior admission burden, congestive heart failure, inflammatory markers, albumin, and time to NIV were broadly similar between outcome groups. Detailed values and component events are presented in Table 2 and Supplementary Table S10.

**Table 2:** Baseline and pre-NIV characteristics according to NIV outcome.

Characteristic	Overall (*n* = 924)	NIV success (*n* = 737)	NIV failure (*n* = 187)	*p* value
Age, years	72.1 ± 9.1	72.0 ± 9.1	72.3 ± 9.5	0.716
Male sex	658 (71.2%)	521 (70.7%)	137 (73.3%)	0.488
Body mass index, kg/m^2^	21.9 ± 3.2	21.9 ± 3.2	21.7 ± 3.2	0.307
≥2 AECOPD admissions in previous year	414 (44.8%)	323 (43.8%)	91 (48.7%)	0.235
Home oxygen therapy	203 (22.0%)	149 (20.2%)	54 (28.9%)	0.011
Congestive heart failure	123 (13.3%)	94 (12.8%)	29 (15.5%)	0.322
Pneumonia-triggered exacerbation	248 (26.8%)	177 (24.0%)	71 (38.0%)	<0.001
Heart rate before NIV, beats/min	109.0 ± 11.4	108.0 ± 11.2	113.4 ± 11.3	<0.001
Respiratory rate before NIV, breaths/min	30.6 ± 4.8	30.0 ± 4.5	33.2 ± 5.0	<0.001
Arterial pH before NIV	7.276 ± 0.033	7.279 ± 0.033	7.264 ± 0.033	<0.001
PaCO2 before NIV, mmHg	74.0 ± 9.9	73.1 ± 9.9	77.5 ± 8.8	<0.001
C-reactive protein, mg/L	23.6 (16.1–33.5)	23.6 (16.1–34.3)	23.8 (16.2–32.0)	0.887
Albumin, g/L	35.4 ± 3.2	35.5 ± 3.2	35.1 ± 3.0	0.071
Time from arrival to NIV, h	3.3 (1.9–5.4)	3.4 (1.9–5.4)	3.1 (1.9–5.2)	0.497

Data are mean ± standard deviation, median (interquartile range), or *n* (%). *p* values compare NIV success and NIV failure. AECOPD, acute exacerbation of chronic obstructive pulmonary disease; NIV, noninvasive ventilation; PaCO₂, arterial partial pressure of carbon dioxide.

### 3.3 Primary dynamic model

After multivariable adjustment, respiratory rate and level of consciousness emerged as the clearest prognostic signals. Faster respiratory rate before NIV and persistent tachypnea at reassessment were associated with greater failure risk, whereas better consciousness at reassessment was associated with lower risk. The association with GCS was the strongest among the displayed predictors, supporting the clinical importance of neurological recovery during correction of hypercapnia. Pneumonia and oxygenation showed clinically plausible but imprecise directions, while pH did not retain an independent association after respiratory rate, consciousness, gas exchange, and the other covariates were considered. These findings indicate overlap among physiological domains rather than absence of clinical relevance for acidosis or oxygenation (Table 3; Supplementary Table S2). The complete equation and calculator are provided in Supplementary Table S9.

**Table 3:** Selected coefficients from the primary dynamic logistic regression model in the development cohort.

Predictor	Adjusted OR	95% CI	*p* value
Pneumonia-triggered exacerbation	1.45	0.89 to 2.35	0.131
Respiratory rate before NIV, per breath/min	1.05	1.00 to 1.11	0.044
Respiratory rate at 2 h, per breath/min	1.06	1.01 to 1.11	0.032
Glasgow Coma Scale at 2 h, per point	0.48	0.35 to 0.65	<0.001
Arterial pH at 2 h, per 0.01-unit increase	0.99	0.91 to 1.08	0.825
PaO2/FiO2 at 2 h, per 10-unit increase	0.95	0.89 to 1.01	0.077

The model included all predictors listed in Supplementary Table S2. Arterial pH is expressed per 0.01-unit increase and PaO₂/FiO₂ per 10-unit increase. CI, confidence interval; GCS, Glasgow Coma Scale; NIV, noninvasive ventilation; OR, odds ratio.

### 3.4 Discrimination, calibration, and classification

**Figure 2:**
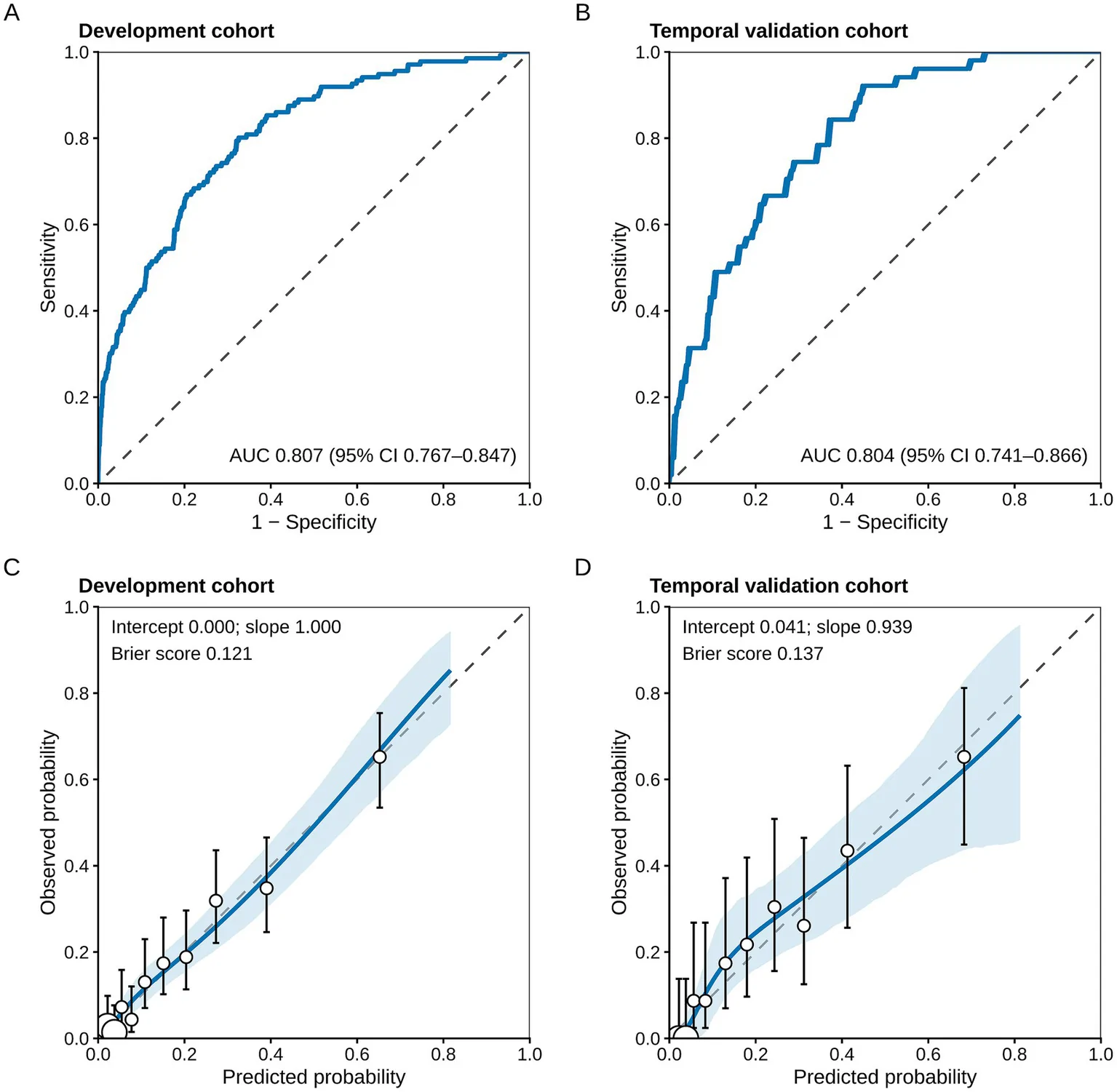
Discrimination and calibration of the primary dynamic model. Panels **(A,B)** show receiver operating characteristic curves in the development and temporal validation cohorts. Panels **(C,D)** show flexible calibration curves with 1,000-resample nonparametric bootstrap pointwise 95% confidence bands. Grouped circles show observed NIV-failure proportions with Wilson 95% confidence intervals; circle size is proportional to the number of patients in each group. The dashed diagonal indicates perfect calibration.

The dynamic model retained good discrimination and close agreement between predicted and observed risk in temporal validation. Its validation AUC was 0.804, with a calibration intercept of 0.041, calibration slope of 0.939, and Brier score of 0.137. The pre-NIV model and the development-recalibrated HACOR probability had numerically lower discrimination and less favorable overall prediction error. However, the AUC differences were small and paired comparisons did not establish superiority of the dynamic model. Thus, the main distinction was a reproducible continuous probability estimate rather than a demonstrated replacement for HACOR (Table 1; Figure 2).

Classification performance varied substantially with the selected threshold. Lower thresholds identified more failures but labeled more successful patients as high risk, whereas higher thresholds increased specificity at the cost of sensitivity. These operating points are action dependent and were not interpreted as validated treatment cutoffs. Detailed classification results for the dynamic model and comparators are reported in Supplementary Table S13.

### 3.5 Decision-curve and clinical-impact analyses

**Figure 3:**
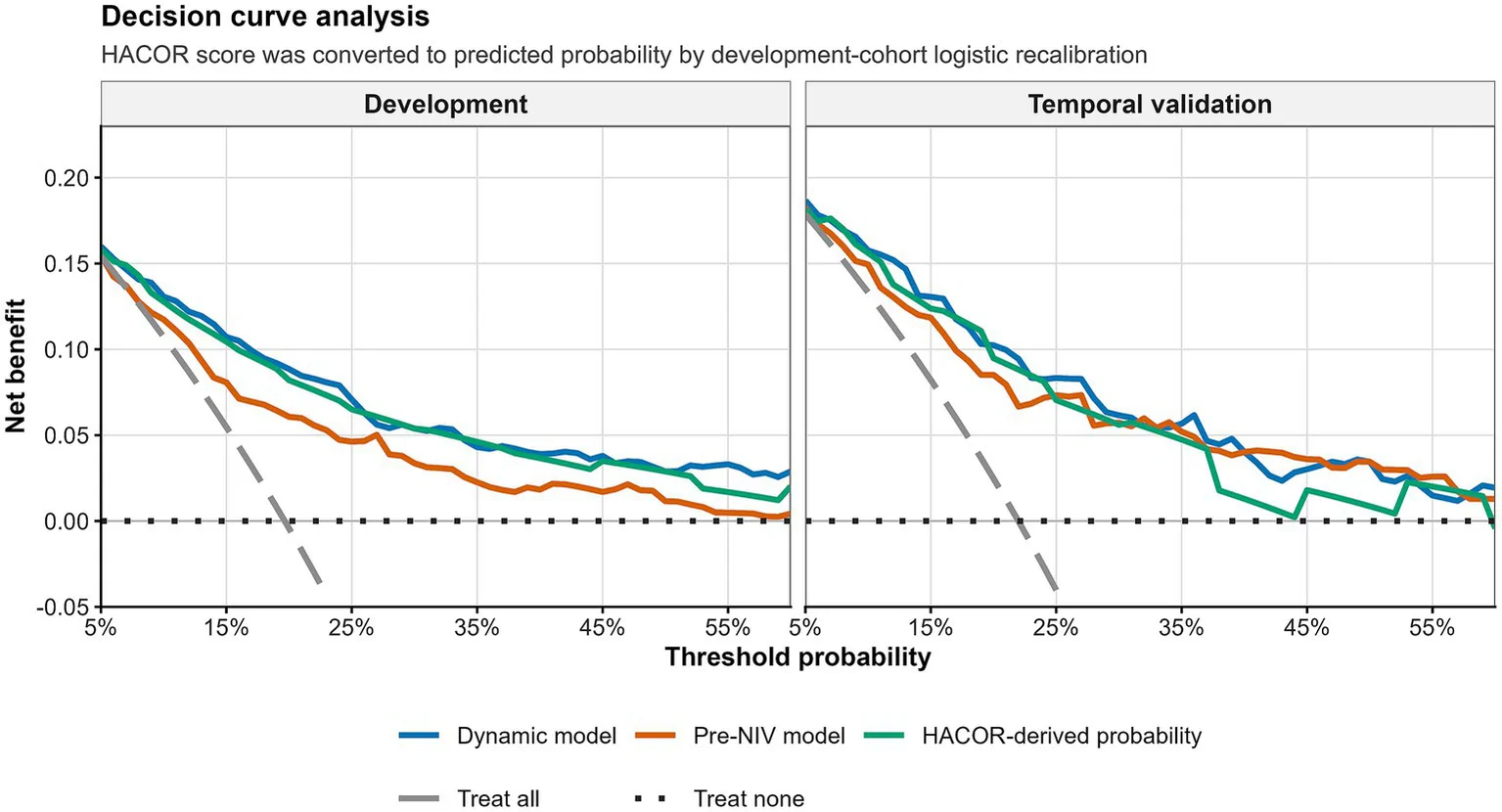
Decision-curve analysis. Net benefit is shown for the primary dynamic model, pre-NIV model, HACOR-derived predicted probability, treat-all strategy, and treat-none strategy in the development and temporal validation cohorts. Raw HACOR was converted to probability using a logistic recalibration model fitted in the development cohort and applied unchanged in validation. The curves describe potential use for intensified reassessment and do not define an automatic intubation threshold.

**Figure 4:**
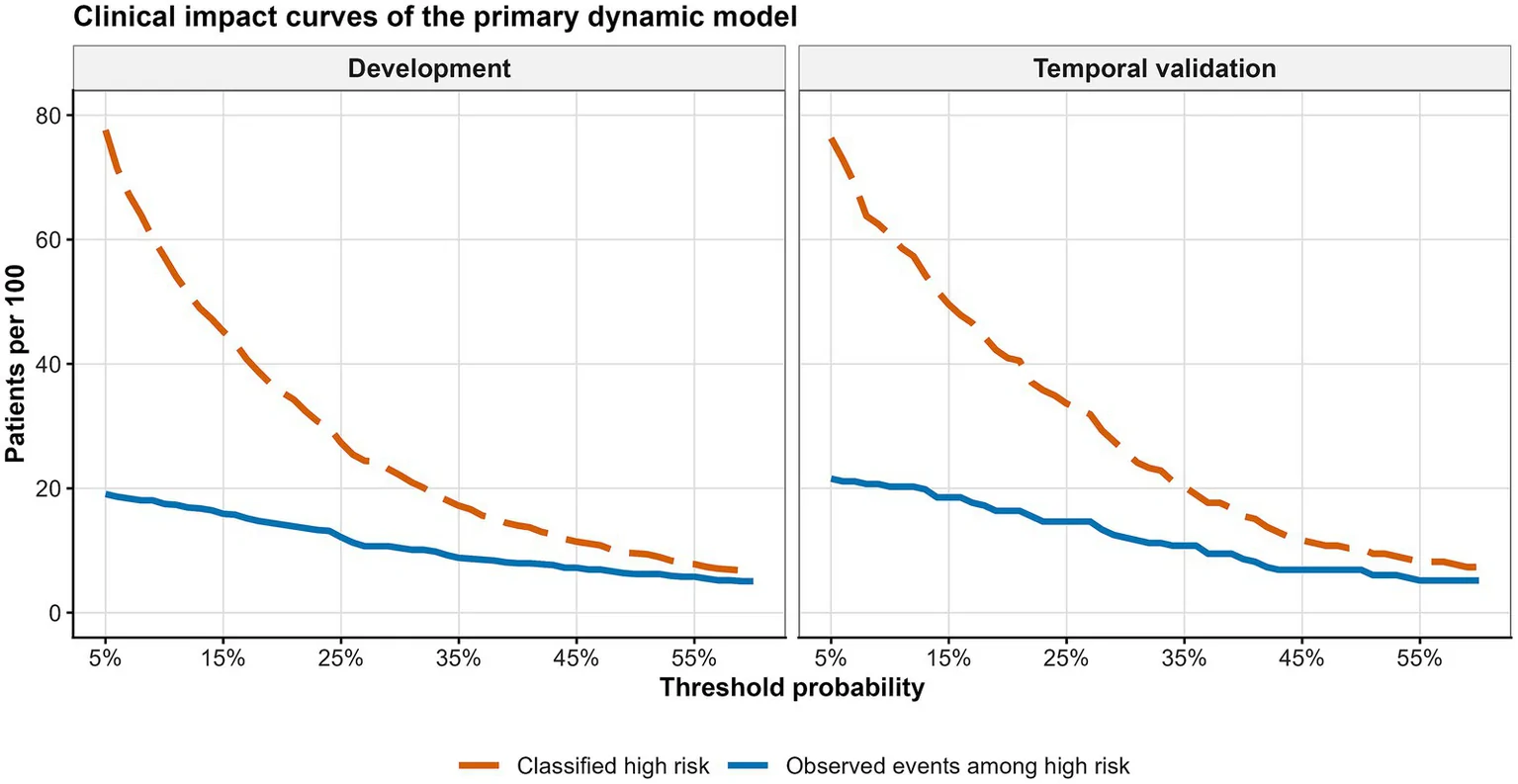
Clinical impact curves for the primary dynamic model. For each threshold probability, the curves show the number classified as high risk per 100 patients and the number of observed NIV failures among patients classified as high risk in the development and temporal validation cohorts.

Decision-curve analysis showed only modest separation among the dynamic, pre-NIV, and HACOR-derived probability curves in temporal validation. The clinical-impact curves showed that patients classified as high risk outnumbered the failures observed within that group, illustrating the false-positive burden expected when the model is used to intensify surveillance rather than to diagnose failure (Figures 3, 4).

### 3.6 Sensitivity and robustness analyses

Results were directionally consistent when the outcome was restricted to intubation or death, intubation alone, early failure, and the cohort without pneumonia-triggered exacerbation. No clear departure from the prespecified functional forms was detected, and inverse-probability weighting yielded similar performance. ICU-transfer-only events remained a specific limitation because the records could not reliably determine whether NIV continued and ultimately succeeded after transfer (Supplementary Tables S3, S10–S12).

Penalized analyses clarified the relation between model complexity and calibration. Stronger one-standard-error penalties preserved ranking but compressed the range of predicted risks, whereas minimum-deviance penalties reduced this over-shrinkage without producing a consistent overall advantage over the primary model. Elastic-net and reduced-model analyses concentrated much of the prognostic information in respiratory rate, consciousness, and oxygenation at reassessment. Nevertheless, models restricted to those variables showed less favorable temporal-validation discrimination, calibration, and overall prediction error than the full prespecified model. The reduced specifications were therefore treated as exploratory candidates for future external validation rather than *post hoc* replacements. Bootstrap correction indicated residual optimism despite the descriptive sample-size assessment (Supplementary Tables S4–S7, S14–S15; Supplementary Figure S1).

## 4 Discussion

The central clinical finding is that physiological response after NIV begins provides prognostic information that is not fully captured by presentation severity. NIV is both a treatment and a brief physiological challenge: effective unloading should reduce respiratory distress, improve alveolar ventilation, and allow mental status to recover. A patient who remains tachypneic, encephalopathic, or otherwise unstable despite support is following a different trajectory from a patient whose work of breathing and consciousness improve. The model is therefore best understood as a structured reassessment aid for patients who remain candidates for an ongoing NIV trial, not as an initiation-time predictor or a substitute for immediate escalation when conventional warning signs are already present (32, 33).

Respiratory rate was the most consistent physiological signal across baseline and reassessment. Persistent tachypnea integrates several processes that matter clinically: high ventilatory demand, respiratory muscle loading, dynamic hyperinflation, distress, ineffective patient–ventilator interaction, and progression of the precipitating illness. Its presence before treatment reflects the initial burden of respiratory failure, whereas persistence after support suggests that unloading or disease reversal is incomplete. Respiratory rate is attractive because it is inexpensive, repeatable, and available in almost every care setting. It is not specific, however, and may also be influenced by pain, anxiety, fever, metabolic acidosis, sedative withdrawal, or observer technique. It should therefore trigger reassessment of the whole clinical picture rather than be interpreted in isolation (10, 14, 34, 35).

Level of consciousness carried the strongest adjusted association at reassessment and has a direct pathophysiological link to hypercapnic respiratory failure. Carbon dioxide retention can depress cerebral function, and successful improvement in ventilation may be followed by recovery of alertness. Failure to regain consciousness may indicate persistent ventilatory insufficiency, a more severe precipitating illness, reduced physiological reserve, or an alternative neurological or metabolic process. This is consistent with the prominence of consciousness in HACOR and previous COPD cohorts. At the same time, GCS is not a pure measure of hypercapnia: sedating medication, delirium, neurologic disease, fatigue, sleep deprivation, and documentation practice can affect the recorded score. Clinical use should therefore distinguish reversible hypercapnic encephalopathy from other causes of impaired responsiveness (16, 17).

Acidosis and oxygenation require more contextual interpretation. Arterial pH remains central to the diagnosis and management of acute hypercapnic respiratory failure, but in a multivariable model its information may overlap with respiratory rate, consciousness, and carbon dioxide burden. A weakened independent association does not imply that acidosis is unimportant for care; it indicates that several variables describe related aspects of the same physiological state. Oxygenation is similarly complex. It reflects lung pathology and ventilation–perfusion mismatch, but is also affected by oxygen delivery, interface leak, device configuration, pneumonia, secretion burden, and the timing of arterial sampling. These dependencies explain why oxygenation may contribute to risk ranking without behaving as a stable isolated determinant across settings (8, 13).

Pneumonia provides a further example of the difference between clinical relevance and independent model contribution. In a patient with AECOPD, pneumonia can add shunt, inflammatory burden, secretions, fever, and greater ventilatory demand, all of which can reduce the likelihood of an uncomplicated NIV response. Yet pneumonia-triggered exacerbations are heterogeneous, and radiographic interpretation, treatment location, microbiology, and illness severity vary considerably. Its prognostic meaning is therefore likely to depend on the surrounding physiological phenotype rather than operate as a uniform binary effect. The sensitivity analysis excluding pneumonia supports the view that the model was not simply identifying this subgroup, while the imprecise adjusted association argues against presenting pneumonia as a standalone determinant (36).

Baseline and reassessment variables answer related but different clinical questions. Baseline factors describe vulnerability before support begins, including recent exacerbation burden, chronic oxygen dependence, comorbidity, the precipitating illness, and the initial physiological disturbance. Reassessment variables describe whether that burden is reversible under treatment. The dominance of dynamic variables therefore does not make the baseline information irrelevant; rather, baseline context can modify the meaning of the observed response. Similar respiratory rates after treatment may imply different risks in patients with different reserve, precipitating causes, or delays before support. This distinction is important when considering whether a future simplified model should use only treatment-response measurements or retain selected contextual variables.

The reduced-model analyses reinforce the biological premise that early treatment response contains most of the readily interpretable signal. They also show why a parsimonious bedside tool is attractive: respiratory rate, consciousness, pH, and oxygenation are already part of routine reassessment and can be obtained without specialized testing. However, simplification involves a trade-off. The full prespecified model combined the dynamic response with baseline burden and clinical context, and this broader information improved overall validation performance. The current data do not justify replacing it through data-driven variable selection. A simpler reassessment model should instead be specified prospectively and tested in an independent multicenter cohort, where ease of use, missingness, transportability, and calibration can be compared directly with the full equation.

Comparison with HACOR should remain conservative. HACOR is faster, simpler, and familiar to many clinicians, whereas the proposed model requires more inputs and a calculator. The present study does not establish superiority or replacement of HACOR. The potential distinction is that a logistic model yields a continuous probability that can be linked to graded responses, while a point score is usually interpreted through broad risk categories. Even this theoretical advantage is clinically meaningful only if probability estimates are well calibrated in the intended population and connected to a clearly defined action. Prospective evaluation is needed to determine whether the added complexity improves the timing, consistency, or appropriateness of reassessment beyond a well-implemented HACOR-based pathway (17, 27, 28, 37).

The threshold and decision-curve findings support an action-specific rather than universal interpretation. The acceptable balance between missed deterioration and unnecessary escalation depends on what follows a positive result. A lower threshold may be reasonable for closer bedside observation, repeat blood-gas testing, or earlier senior review because these actions carry limited harm. The same threshold would be inappropriate if it automatically prompted invasive ventilation or ICU transfer. Decision curves can describe the potential net benefit of a risk-guided strategy under specified preferences, but they do not define the intervention or prove that clinicians will act consistently. Real-world variation in patient selection, monitoring intensity, staffing, and escalation practice makes a standardized response pathway essential before impact testing (38, 39).

Practical implementation would therefore require more than distributing the equation or calculator. The reassessment time, measurement conditions, and required inputs would need to be standardized; missing or implausible values would need explicit handling; and the output would need to be embedded in a workflow that specifies who reviews the estimate and what actions are considered. The model should complement bedside examination, trajectory assessment, secretion management, patient tolerance, and goals of care. A probability estimate that is technically reproducible but disconnected from a clinical response can add documentation burden without improving outcomes. Human-factors testing and prospective evaluation of adherence, clinician override, and unintended escalation are essential before routine use.

The composite outcome captures clinically important deterioration but has limited face validity as a direct measure of failure of the NIV modality. Endotracheal intubation, death, and transfer to a higher-acuity location are consequential events, yet they arise through different clinical and organizational processes. In particular, some patients may be transferred to an ICU for closer monitoring while NIV is continued and ultimately succeeds. The harder-endpoint analyses reduce concern that transfer practice alone generated the observed signal, but they cannot determine the reason for every escalation. Future studies should prospectively adjudicate physiological failure, interface intolerance, secretion management, hemodynamic deterioration, treatment limitations, and organizational transfer decisions.

Methodologically, the study preserves the clinical sequence of predictor availability, uses temporal validation rather than a random same-period split, evaluates calibration and overall prediction error, and provides a complete equation and calculator (40, 41). Functional-form, weighting, penalized, reduced-model, threshold, and endpoint analyses improve transparency. At the same time, penalization and bootstrap correction show that prediction range and optimism are sensitive to model complexity and sample size. A descriptive sample-size criterion cannot guarantee stable calibration, and a later-period cohort from the same institution remains closer to the development setting than a genuinely external population. These findings support cautious transport within the local care pathway rather than broad claims of generalizability (20, 22, 23, 29–31, 42).

Several limitations define the next research steps. The single cancer-specialty setting may differ from general respiratory units in case mix, monitoring, and thresholds for ICU transfer or intubation. Measurements were retrospectively harmonized rather than collected under a uniform protocol, detailed cancer status and interface information were unavailable, and records with unresolved endpoint inconsistencies were excluded. The available variables also did not support a formal assessment of performance or fairness across broader sociodemographic groups. The landmark design conditions on patients who remain alive and free from immediate escalation until reassessment, so the model does not represent the most rapidly deteriorating patients. Finally, the study evaluates predictive performance rather than clinical impact. The appropriate next step is multicenter external validation of the unchanged equation, followed by prospective comparison of the full and parsimonious approaches within a prespecified reassessment pathway that allows clinician override and measures both patient outcomes and resource consequences.

## 5 Conclusion

Early physiological reassessment after NIV identifies clinically meaningful prognostic information, particularly persistent tachypnea and impaired recovery of consciousness. The full model provides a reproducible probability estimate but has not shown clear superiority over HACOR and should not direct NIV initiation or autonomous escalation. Independent multicenter validation, comparison with simpler reassessment strategies, and prospective impact evaluation are required before routine clinical use.

## Data Availability

The original contributions presented in the study are included in the article/Supplementary material, further inquiries can be directed to the corresponding author.
