# Supplementary material for: Two-hour dynamic reassessment of noninvasive ventilation failure risk in acute exacerbation of chronic obstructive pulmonary disease with hypercapnic respiratory failure: development and temporal validation
Source: Front Med (Lausanne). 2026 Aug 14;13:1920677. doi: 10.3389/fmed.2026.1920677 (PMC13522161; doi:10.3389/fmed.2026.1920677)
Supplement: Supplementary file 1 [file Supplementary_file_1.docx]

**Completed TRIPOD+AI Checklist**

**Two-Hour Dynamic Reassessment of Noninvasive Ventilation Failure Risk in AECOPD With Hypercapnic Respiratory Failure**

Page references correspond to the clean balanced revised manuscript. Supplementary references are identified explicitly. D = model development; E = model evaluation.

| **Section / topic** | **Item** | **D / E** | **TRIPOD+AI checklist item** | **Reported on page / section** |
| --- | --- | --- | --- | --- |
| TITLE | 1 | D;E | Identify the study as developing or evaluating the performance of a multivariable prediction model, the target population, and the outcome to be predicted. | Title page, p1. |
| ABSTRACT | 2 | D;E | See TRIPOD+AI for Abstracts checklist. | Structured abstract, p2. |
| INTRODUCTION - Background | 3a | D;E | Explain the healthcare context (including whether diagnostic or prognostic) and rationale for developing or evaluating the prediction model, including references to existing models. | Introduction, pp2-4. |
| INTRODUCTION - Background | 3b | D;E | Describe the target population and the intended purpose of the prediction model in the context of the care pathway, including its intended users. | Introduction, pp2-4; intended use reiterated in Discussion, pp8-11. |
| INTRODUCTION - Background | 3c | D;E | Describe any known health inequalities between sociodemographic groups. | Not specifically evaluated. Detailed sociodemographic attributes beyond age and sex were not consistently captured; the inability to assess fairness or subgroup performance is stated in Discussion, p11. |
| INTRODUCTION - Objectives | 4 | D;E | Specify the study objectives, including whether the study describes development or validation of a prediction model (or both). | Final paragraph of Introduction, p4. |
| METHODS - Data | 5a | D;E | Describe the sources of data separately for the development and evaluation datasets, the rationale for using these data, and representativeness of the data. | Methods 2.1, p4. |
| METHODS - Data | 5b | D;E | Specify dates of collected participant data, including start and end of accrual and, if applicable, end of follow-up. | Methods 2.1, p4. |
| METHODS - Participants | 6a | D;E | Specify key elements of the study setting, including number and location of centres. | Methods 2.1, p4. |
| METHODS - Participants | 6b | D;E | Describe eligibility criteria for study participants. | Methods 2.2, p4; intended-use spectrum reiterated in Discussion, pp8-11. |
| METHODS - Participants | 6c | D;E | Give details of treatments received and how they were handled during development or evaluation, if relevant. | Methods 2.3, pp4-5. |
| METHODS - Data preparation | 7 | D;E | Describe data preprocessing and quality checking, including whether this was similar across relevant sociodemographic groups. | Methods 2.2-2.3, pp4-5; Supplementary Methods, p1. No sociodemographic-group-specific preprocessing was performed. |
| METHODS - Outcome | 8a | D;E | Clearly define the predicted outcome and time horizon, including how and when assessed, rationale, and whether assessment was consistent across sociodemographic groups. | Methods 2.4, p5; Supplementary Tables S10A-S10C, p6. |
| METHODS - Outcome | 8b | D;E | If outcome assessment requires subjective interpretation, describe qualifications and demographic characteristics of outcome assessors. | Outcome was derived from routine clinical endpoint documentation rather than a separate study-specific assessor; assessor demographic characteristics were not captured. Methods 2.2 and 2.4, pp4-5. |
| METHODS - Outcome | 8c | D;E | Report actions to blind assessment of the outcome to be predicted. | Retrospective routine-care endpoint extraction was used; no study-specific blinded outcome adjudication was performed. Methods 2.2 and 2.4, pp4-5. |
| METHODS - Predictors | 9a | D | Describe choice of initial predictors and any preselection before model building. | Methods 2.5, p5. |
| METHODS - Predictors | 9b | D;E | Clearly define all predictors, including how and when measured and any actions to blind predictor assessment. | Methods 2.3 and 2.5, pp4-5; Supplementary Table S9, p6. |
| METHODS - Predictors | 9c | D;E | If predictor measurement requires subjective interpretation, describe qualifications and demographic characteristics of predictor assessors. | GCS and other clinical measurements were recorded during routine care; study-specific assessor demographics were not captured. Methods 2.3, pp4-5. |
| METHODS - Sample size | 10 | D;E | Explain how study size was arrived at separately for development and evaluation, justify sufficiency, and include details of sample-size calculation. | Methods 2.10, p6; Results 3.6, p8; Discussion, p11; Supplementary Tables S15A-S15B, p11. |
| METHODS - Missing data | 11 | D;E | Describe handling of missing data and reasons for omitting data. | Methods 2.2, p4, and 2.10, p6; Supplementary Methods, p1. |
| METHODS - Analytical methods | 12a | D | Describe how data were used for development and evaluation, including partitioning and sample-size considerations. | Methods 2.1, p4, and 2.9-2.10, p6. |
| METHODS - Analytical methods | 12b | D | Describe handling of predictors in analyses, including functional form, rescaling, transformation, or standardisation. | Methods 2.5, p5; Supplementary Table S11, p7. |
| METHODS - Analytical methods | 12c | D | Specify model type, rationale, model-building steps, hyperparameter tuning, and internal validation. | Methods 2.5, p5, and 2.9, p6; Supplementary Tables S4-S7, pp4-5. |
| METHODS - Analytical methods | 12d | D;E | Describe handling and quantification of heterogeneity across clusters. | Not applicable: single-centre study without a multilevel cluster structure. Methods 2.1, p4; generalisability limitation, p11. |
| METHODS - Analytical methods | 12e | D;E | Specify all measures and plots used to evaluate model performance and compare models. | Methods 2.7-2.9, pp5-6. |
| METHODS - Analytical methods | 12f | E | Describe any model updating arising from model evaluation. | HACOR logistic recalibration: Methods 2.6, p5; Results 3.4, p7; Table 3, p19. |
| METHODS - Analytical methods | 12g | E | For model evaluation, describe how predictions were calculated. | Methods 2.5-2.6, p5; complete equation and coding in Supplementary Table S9, p6; offline calculator. |
| METHODS - Class imbalance | 13 | D;E | If class-imbalance methods were used, state why and how, and any subsequent recalibration. | No class-imbalance resampling, synthetic sampling, or outcome weighting was used; models used the observed outcome distribution. Methods 2.9, p6. |
| METHODS - Fairness | 14 | D;E | Describe approaches used to address model fairness and their rationale. | No formal fairness analysis was undertaken because detailed sociodemographic attributes beyond age and sex were not consistently captured. Discussion limitation, p11. |
| METHODS - Model output | 15 | D | Specify model output and provide rationale for classification and threshold identification. | Methods 2.5 and 2.7, p5; Table 3, p19; Supplementary Table S13, p9. |
| METHODS - Training versus evaluation | 16 | D;E | Identify differences between development and evaluation data in setting, eligibility, outcome, and predictors. | Methods 2.1, p4; Results 3.1, pp6-7; Supplementary Table S1, p2. |
| METHODS - Ethical approval | 17 | D;E | Name ethics committee and describe informed consent or waiver. | Methods 2.11, p6; Ethics statement, p11. |
| OPEN SCIENCE - Funding | 18a | D;E | Give funding source and role of funders. | Funding statement, p12. |
| OPEN SCIENCE - Conflicts of interest | 18b | D;E | Declare conflicts of interest and financial disclosures for all authors. | Conflict of interest statement, p12. |
| OPEN SCIENCE - Protocol | 18c | D;E | Indicate where the study protocol can be accessed or state that a protocol was not prepared. | The ethics-approved protocol is described in Methods 2.11, p6. No public protocol repository location was available. |
| OPEN SCIENCE - Registration | 18d | D;E | Provide registration information or state that the study was not registered. | Methods 2.11, p6: the study was not prospectively registered. |
| OPEN SCIENCE - Data sharing | 18e | D;E | Provide details of availability of study data. | Data availability statement, p11. |
| OPEN SCIENCE - Code sharing | 18f | D;E | Provide details of availability of analytical code. | Methods 2.10, p6; Data availability statement, p11; submitted R scripts. |
| PATIENT & PUBLIC INVOLVEMENT | 19 | D;E | Provide details of patient/public involvement or state no involvement. | Methods 2.11, p6: patients and members of the public were not involved in the design, conduct, reporting, or dissemination planning of this retrospective study. |
| RESULTS - Participants | 20a | D;E | Describe participant flow, numbers with/without outcome, and follow-up if applicable. | Results 3.1, pp6-7; Figure 1 legend, p16. |
| RESULTS - Participants | 20b | D;E | Report participant characteristics, dates, predictors, treatments, sample size, events, follow-up, missing data, and demographic differences. | Results 3.1-3.2, pp6-7; Table 1, pp17-18; Supplementary Tables S1 and S8, pp2 and 5. |
| RESULTS - Participants | 20c | E | For evaluation, compare distributions of important predictors and outcomes with development data. | Results 3.1, pp6-7; Supplementary Table S1, p2. |
| RESULTS - Model development | 21 | D;E | Specify numbers of participants and events in each analysis. | Results 3.1 and 3.6, pp6-8; Table 3, p19; Supplementary Tables S3-S5, S12, and S14-S15, pp3-4 and 7-11. |
| RESULTS - Model specification | 22 | D | Provide full prediction model to allow individual predictions and third-party evaluation/implementation, including restrictions. | Supplementary Table S9, p6; offline risk calculator; Data availability statement, p11. |
| RESULTS - Model performance | 23a | D;E | Report performance estimates with confidence intervals, including key subgroups where relevant; consider plots. | Results 3.4-3.6, pp7-8; Table 3, p19; Figures 2-4; Supplementary Tables S3-S6, S12, and S14, pp3-4, 7-8, and 10. |
| RESULTS - Model performance | 23b | D;E | If examined, report heterogeneity in performance across clusters. | Not applicable: single-centre study; between-centre heterogeneity could not be estimated. |
| RESULTS - Model updating | 24 | E | Report results from model updating, including updated model and subsequent performance. | Development-derived HACOR probability and validation performance: Results 3.4, p7; Table 3, p19. |
| DISCUSSION - Interpretation | 25 | D;E | Give overall interpretation of results, including fairness, in context of objectives and previous studies. | Discussion, pp8-11; fairness limitation, p11. |
| DISCUSSION - Limitations | 26 | D;E | Discuss limitations and effects on bias, uncertainty, and generalisability. | Discussion limitations, p11. |
| DISCUSSION - Usability | 27a | D | Describe how poor-quality or unavailable input data should be assessed and handled in implementation. | Methods 2.3 and 2.10, pp4-6; Supplementary Table S9 and calculator; Discussion implementation and limitations, pp10-11. |
| DISCUSSION - Usability | 27b | D | Specify required user interaction and expertise for input handling and model use. | Introduction intended use, pp2-4; Discussion and Conclusion, pp8-11; calculator instructions. |
| DISCUSSION - Usability | 27c | D;E | Discuss next research steps with specific view to applicability and generalisability. | Discussion limitations and future research, pp10-11; Conclusion, p11. |

*Checklist source: Collins GS, Moons KGM, Dhiman P, et al. TRIPOD+AI statement: updated guidance for reporting clinical prediction models that use regression or machine learning methods. BMJ. 2024;385:e078378.*
