# Supplementary material for: Two-hour dynamic reassessment of noninvasive ventilation failure risk in acute exacerbation of chronic obstructive pulmonary disease with hypercapnic respiratory failure: development and temporal validation
Source: Front Med (Lausanne). 2026 Aug 14;13:1920677. doi: 10.3389/fmed.2026.1920677 (PMC13522161; doi:10.3389/fmed.2026.1920677)
Supplement: Supplementary file 2 [file Supplementary_file_2.docx]

# Supplementary methods

## Data-consistency rules applied before analysis

The source dataset contained 1,040 records with unique patient identifiers, and only the first eligible hospitalization per patient was retained. Before analysis, predefined consistency checks were applied. A record was excluded when the coded NIV-failure status disagreed with any of the intubation, ICU-transfer, or in-hospital-death fields; when a coded failure lacked a recorded failure time; or when ICU length of stay exceeded total hospital length of stay. The exclusions comprised 104 records with endpoint inconsistency and 24 with impossible length-of-stay values, including 12 records that met both criteria, leaving 924 patients for analysis. Records with unresolved inconsistencies were excluded without imputation or retrospective recoding.

## Model equations and reproducibility

The pre-NIV and dynamic logistic-regression coefficients were estimated in the 2021-2024 development cohort. Predicted probability was calculated as p = 1/[1 + exp(-LP)], where LP is the intercept plus the sum of each coefficient multiplied by its predictor value. Complete coefficients and the dynamic-model equation are reported in Supplementary Table S9. The HACOR probability used for probability-based performance evaluation and decision-curve analysis was generated with logit(p) = -3.5508 + 0.3029 × HACOR at 2 hours. The equation was fitted in development and applied unchanged in temporal validation. The accompanying spreadsheet calculator applies the coefficient values reported in Supplementary Table S9.

## Analysis unit, intended-use population, and reassessment timing

Only the first hospitalization meeting all eligibility criteria was retained for each patient. The 1,040 source records contained 1,040 unique patient identifiers. The intended-use population comprised patients who remained alive, had not undergone intubation, and had not otherwise met immediate escalation criteria at the first complete reassessment obtained 90-150 minutes after documented NIV initiation. When more than one complete assessment was available, the assessment closest to 120 minutes was selected. Arterial blood-gas and bedside physiological measurements were required to originate from the same assessment episode.

## Oxygen-record harmonization and excluded pre-NIV oxygenation field

Arterial blood-gas values were paired with the contemporaneous oxygen record. FiO₂ came from the NIV-device integrated blender display in 771 of 924 patients (83.4%), a fixed setting in the contemporaneous respiratory-therapy record in 92 (10.0%), and a device-specific estimate from oxygen bleed-in in 61 (6.6%). Respiratory-therapy entries were accepted when recorded within 10 minutes of blood-gas sampling. Measurements were considered relatively stable when NIV remained in place and the interface, inspiratory pressure, expiratory pressure, and oxygen setting had not materially changed during the preceding 20 minutes. This was retrospective operational harmonization rather than protocol-standardized sampling. NIV interface type was not consistently captured. The pre-NIV PaO₂/FiO₂ field available in the original extract was excluded because its timestamp and FiO₂ provenance could not be reconciled with the separately exported PaO₂ and FiO₂ measurements.

## Outcome, performance, and additional analyses

The primary outcome was the documented composite of endotracheal intubation, ICU transfer, or in-hospital death after NIV initiation. ICU transfer alone was not assumed to establish physiological failure of NIV. Sensitivity analyses therefore evaluated endotracheal intubation or in-hospital death, intubation alone, failure within 24 hours, and exclusion of pneumonia-triggered exacerbations. ICU-transfer-only patterns were summarized separately.

Discrimination was assessed by AUC, calibration by intercept, slope, plots with bootstrap bands and grouped Wilson intervals, and overall prediction error by Brier score. Classification was evaluated at development-derived and fixed probability thresholds. Decision-curve and clinical-impact analyses evaluated potential use for intensified reassessment rather than automatic intubation. Each continuous predictor was assessed for departure from linearity. Selection associated with exclusions was examined by inverse-probability-of-inclusion weighting. Ridge and elastic-net models used identical outcome-stratified cross-validation folds, and performance was reported at one-standard-error and minimum-deviance penalties. Exploratory reduced models tested the information carried by the reassessment variables. A descriptive Riley-framework assessment evaluated development sample size; full analytic specifications are reflected in Supplementary Tables S3-S15.

# Supplementary results

## Cohort assembly and clinical associations

After prespecified consistency checks, 924 patients remained from 1,040 source records. The development and temporal-validation cohorts included 692 and 232 patients, with 136 and 51 NIV-failure events, respectively. Patients with failure more often had home oxygen use and pneumonia-triggered exacerbation and showed faster heart and respiratory rates, lower pH, and higher PaCO₂ before NIV. Detailed distributions are provided in Supplementary Tables S1, S8, and S10.

## Primary model and comparator performance

The dynamic model retained temporal-validation discrimination and calibration close to its development performance. The pre-NIV model and development-recalibrated HACOR probability had lower numerical performance, but paired discrimination comparisons did not establish superiority of the dynamic model. Detailed model performance, calibration, classification, and threshold trade-offs are reported in Supplementary Tables S4-S6 and S13-S14.

## Robustness, parsimony, and sample size

Results were consistent across harder endpoints, early failure, exclusion of pneumonia-triggered exacerbations, functional-form assessment, and inverse-probability weighting. Strong one-standard-error penalization compressed predicted risks, whereas minimum-deviance penalties reduced over-shrinkage. Reassessment variables carried most of the readily interpretable signal, but exploratory reduced models performed less well overall than the full model. Bootstrap correction indicated residual optimism despite the development cohort exceeding the nominal post hoc Riley-framework minimum; detailed results are provided in Supplementary Tables S3-S7 and S11-S15 and Supplementary Figure S1.

# Supplementary tables

**Supplementary Table S1. Comparison of the development and temporal validation cohorts.**

| **Characteristic** | **Development** | **Temporal validation** | **P value** |
| --- | --- | --- | --- |
| Age, years | 72.1 ± 9.3 | 72.1 ± 8.7 | 0.958 |
| Male sex | 485 (70.1%) | 173 (74.6%) | 0.192 |
| Body mass index, kg/m² | 22.0 ± 3.3 | 21.4 ± 3.1 | 0.017 |
| Pneumonia-triggered exacerbation | 192 (27.7%) | 56 (24.1%) | 0.283 |
| Heart rate before NIV, beats/min | 109.1 ± 11.3 | 108.9 ± 11.6 | 0.873 |
| Respiratory rate before NIV, breaths/min | 30.7 ± 4.7 | 30.3 ± 4.9 | 0.222 |
| Arterial pH before NIV | 7.276 ± 0.033 | 7.276 ± 0.033 | 0.705 |
| C-reactive protein, mg/L | 24.4 (16.4-34.1) | 21.7 (15.3-31.8) | 0.091 |
| Heart rate at 2 h, beats/min | 102.6 ± 12.3 | 103.1 ± 11.3 | 0.567 |
| Respiratory rate at 2 h, breaths/min | 30.1 ± 6.3 | 30.6 ± 6.2 | 0.284 |
| Arterial pH at 2 h | 7.293 ± 0.037 | 7.289 ± 0.037 | 0.233 |
| PaO2/FiO2 at 2 h | 200.4 ± 49.9 | 199.1 ± 51.3 | 0.738 |
| NIV failure | 136 (19.7%) | 51 (22.0%) | 0.445 |

Data are mean ± standard deviation, median (interquartile range), or n (%), as appropriate. P values compare the development and temporal validation cohorts.

**Supplementary Table S2. Full coefficient table for the primary dynamic logistic model.**

| **Predictor** | **Adjusted OR** | **95% CI** | **P value** |
| --- | --- | --- | --- |
| ≥2 AECOPD admissions in previous year | 0.86 | 0.55 to 1.33 | 0.495 |
| Home oxygen therapy | 1.02 | 0.60 to 1.70 | 0.951 |
| Congestive heart failure | 1.04 | 0.55 to 1.91 | 0.890 |
| Pneumonia-triggered exacerbation | 1.45 | 0.89 to 2.35 | 0.131 |
| Heart rate before NIV, per beat/min | 1.01 | 0.99 to 1.04 | 0.189 |
| Respiratory rate before NIV, per breath/min | 1.05 | 1.00 to 1.11 | 0.044 |
| Arterial pH before NIV, per 0.01-unit increase | 1.00 | 0.93 to 1.07 | 0.981 |
| PaCO2 before NIV, per mmHg | 1.01 | 0.98 to 1.03 | 0.504 |
| C-reactive protein, per mg/L | 0.99 | 0.97 to 1.00 | 0.092 |
| Albumin, per g/L | 1.00 | 0.93 to 1.08 | 0.974 |
| Time from arrival to NIV, per hour | 0.96 | 0.88 to 1.04 | 0.324 |
| Respiratory rate at 2 h, per breath/min | 1.06 | 1.01 to 1.11 | 0.032 |
| Glasgow Coma Scale at 2 h, per point | 0.48 | 0.35 to 0.65 | <0.001 |
| Arterial pH at 2 h, per 0.01-unit increase | 0.99 | 0.91 to 1.08 | 0.825 |
| PaO2/FiO2 at 2 h, per 10-unit increase | 0.95 | 0.89 to 1.01 | 0.077 |

All predictors were entered simultaneously. Arterial pH variables are expressed per 0.01-unit increase, and PaO₂/FiO₂ at 2 hours per 10-unit increase.

**Supplementary Table S3. Sensitivity analyses of the primary dynamic model.**

| **Analysis** | **Cohort** | **N** | **Events** | **AUC** | **AUC 95% CI** | **Calibration intercept** | **Calibration slope** | **Brier score** |
| --- | --- | --- | --- | --- | --- | --- | --- | --- |
| Primary endpoint: NIV failure composite | Development | 692 | 136 | 0.807 | 0.767 to 0.847 | 0.000 | 1.000 | 0.121 |
| Primary endpoint: NIV failure composite | Temporal validation | 232 | 51 | 0.804 | 0.741 to 0.866 | 0.041 | 0.939 | 0.137 |
| Hard endpoint: endotracheal intubation or in-hospital death | Development | 692 | 112 | 0.787 | 0.743 to 0.831 | 0.000 | 1.000 | 0.114 |
| Hard endpoint: endotracheal intubation or in-hospital death | Temporal validation | 232 | 43 | 0.791 | 0.723 to 0.858 | 0.067 | 1.033 | 0.125 |
| Endotracheal intubation only | Development | 692 | 109 | 0.783 | 0.738 to 0.828 | 0.000 | 1.000 | 0.113 |
| Endotracheal intubation only | Temporal validation | 232 | 43 | 0.784 | 0.714 to 0.854 | 0.102 | 1.038 | 0.127 |
| NIV failure within 24 hours | Development | 692 | 124 | 0.797 | 0.756 to 0.838 | 0.000 | 1.000 | 0.120 |
| NIV failure within 24 hours | Temporal validation | 232 | 47 | 0.792 | 0.727 to 0.857 | 0.062 | 0.939 | 0.135 |
| Primary endpoint after excluding pneumonia-triggered exacerbations | Development | 500 | 86 | 0.795 | 0.746 to 0.845 | 0.000 | 1.000 | 0.117 |
| Primary endpoint after excluding pneumonia-triggered exacerbations | Temporal validation | 176 | 30 | 0.772 | 0.691 to 0.854 | -0.109 | 0.922 | 0.124 |

Each sensitivity model used the same dynamic predictor set, was fitted in development, and was applied unchanged in temporal validation. The hard endpoint combined endotracheal intubation and in-hospital death. Development-cohort calibration intercepts of 0 and slopes of 1 are apparent in-sample values and are not independent evidence of calibration; optimism-corrected and temporal-validation estimates are more informative.

**Supplementary Table S4. Penalized logistic-regression sensitivity analyses using fixed stratified cross-validation folds.**

**Panel A. Penalty selection, model sparsity, and discrimination**

| **Model** | **Penalty** | **λ** | **Nonzero predictors** | **Cohort** | **N** | **Events** | **AUC (95% CI)** |
| --- | --- | --- | --- | --- | --- | --- | --- |
| Ridge logistic regression | One-standard-error | 0.606063 | 15 | Development | 692 | 136 | 0.795 (0.753 to 0.838) |
| Ridge logistic regression | One-standard-error | 0.606063 | 15 | Temporal validation | 232 | 51 | 0.804 (0.740 to 0.868) |
| Ridge logistic regression | Minimum-deviance | 0.078276 | 15 | Development | 692 | 136 | 0.803 (0.761 to 0.844) |
| Ridge logistic regression | Minimum-deviance | 0.078276 | 15 | Temporal validation | 232 | 51 | 0.805 (0.742 to 0.868) |
| Elastic-net logistic regression | One-standard-error | 0.090703 | 3 | Development | 692 | 136 | 0.795 (0.754 to 0.837) |
| Elastic-net logistic regression | One-standard-error | 0.090703 | 3 | Temporal validation | 232 | 51 | 0.772 (0.708 to 0.836) |
| Elastic-net logistic regression | Minimum-deviance | 0.027063 | 6 | Development | 692 | 136 | 0.801 (0.760 to 0.841) |
| Elastic-net logistic regression | Minimum-deviance | 0.027063 | 6 | Temporal validation | 232 | 51 | 0.790 (0.726 to 0.853) |

**Panel B. Calibration and overall prediction error**

| **Model** | **Penalty** | **Cohort** | **Calibration intercept (95% CI)** | **Calibration slope (95% CI)** | **Brier score** |
| --- | --- | --- | --- | --- | --- |
| Ridge logistic regression | One-standard-error | Development | -0.000 (-0.192 to 0.192) | 2.463 (1.984 to 2.942) | 0.134 |
| Ridge logistic regression | One-standard-error | Temporal validation | 0.106 (-0.212 to 0.425) | 2.453 (1.648 to 3.258) | 0.147 |
| Ridge logistic regression | Minimum-deviance | Development | -0.000 (-0.204 to 0.204) | 1.267 (1.025 to 1.510) | 0.123 |
| Ridge logistic regression | Minimum-deviance | Temporal validation | 0.066 (-0.274 to 0.406) | 1.231 (0.828 to 1.633) | 0.136 |
| Elastic-net logistic regression | One-standard-error | Development | 0.000 (-0.193 to 0.193) | 2.114 (1.701 to 2.528) | 0.133 |
| Elastic-net logistic regression | One-standard-error | Temporal validation | 0.082 (-0.239 to 0.403) | 1.712 (1.100 to 2.324) | 0.150 |
| Elastic-net logistic regression | Minimum-deviance | Development | -0.000 (-0.204 to 0.204) | 1.265 (1.022 to 1.508) | 0.124 |
| Elastic-net logistic regression | Minimum-deviance | Temporal validation | 0.059 (-0.282 to 0.400) | 1.097 (0.728 to 1.467) | 0.141 |

Ridge and elastic-net models used the same fixed outcome-stratified 10-fold allocation; elastic net used α = 0.7. The one-standard-error solution applies stronger shrinkage than the minimum-deviance solution. Development results are apparent in-sample estimates. The penalized models are robustness analyses and are not proposed as calibrated alternatives to the prespecified primary model.

**Supplementary Table S5. Bootstrap optimism correction for the primary dynamic model.**

| **Metric** | **Value** |
| --- | --- |
| Apparent AUC | 0.807 |
| Optimism-corrected AUC | 0.781 |
| Apparent calibration slope | 1.000 |
| Optimism-corrected calibration slope | 0.876 |
| Apparent Brier score | 0.121 |
| Optimism-corrected Brier score | 0.129 |

Optimism correction used 1,000 bootstrap resamples from the development cohort.

**Supplementary Table S6. Paired comparisons of discrimination.**

| **Cohort** | **Comparison** | **AUC dynamic** | **AUC comparator** | **Delta AUC** | **Delta AUC 95% CI** | **P value** |
| --- | --- | --- | --- | --- | --- | --- |
| Development | Dynamic model vs pre-NIV model | 0.807 | 0.718 | 0.088 | 0.051 to 0.128 | <0.001 |
| Development | Dynamic model vs HACOR raw score | 0.807 | 0.794 | 0.013 | -0.007 to 0.033 | 0.214 |
| Temporal validation | Dynamic model vs pre-NIV model | 0.804 | 0.758 | 0.046 | -0.017 to 0.110 | 0.154 |
| Temporal validation | Dynamic model vs HACOR raw score | 0.804 | 0.781 | 0.022 | -0.012 to 0.056 | 0.189 |

P values are from paired DeLong tests. Confidence intervals for ΔAUC were estimated with 1,000 paired nonparametric bootstrap resamples.

**Supplementary Table S7. Elastic-net coefficients at the one-standard-error and minimum-deviance penalties.**

| **Predictor** | **One-standard-error β (OR)** | **Minimum-deviance β (OR)** |
| --- | --- | --- |
| At least two AECOPD admissions in the previous year | — | — |
| Home oxygen therapy | — | — |
| Congestive heart failure | — | — |
| Pneumonia-triggered exacerbation | — | — |
| Heart rate before NIV, per beat/min | — | 0.0058 (1.006) |
| Respiratory rate before NIV, per breath/min | — | 0.0315 (1.032) |
| Arterial pH before NIV, per 0.01 unit | — | — |
| PaCO2 before NIV, per mmHg | — | — |
| C-reactive protein, per mg/L | — | — |
| Albumin, per g/L | — | — |
| Time from hospital arrival to NIV, per hour | — | — |
| Respiratory rate at 2 hours, per breath/min | 0.0289 (1.029) | 0.0468 (1.048) |
| Glasgow Coma Scale at 2 hours, per point | -0.4324 (0.649) | -0.6089 (0.544) |
| Arterial pH at 2 hours, per 0.01 unit | — | -0.0107 (0.989) |
| PaO2/FiO2 at 2 hours, per 10-unit increase | -0.0223 (0.978) | -0.0457 (0.955) |

All 15 prespecified predictors were entered simultaneously using α = 0.7 and the same fixed outcome-stratified 10-fold allocation used for ridge regression. An em dash denotes a coefficient of zero. Variable retention is a sensitivity analysis and should not be interpreted as confirmatory predictor selection. OR, odds ratio.

**Supplementary Table S8. Comparison of included records and records excluded before analysis.**

| **Characteristic** | **Included in analysis (n = 924)** | **Excluded before analysis (n = 116)** | **P value** |
| --- | --- | --- | --- |
| Age, years | 72.1 ± 9.1 | 72.1 ± 8.4 | 0.959 |
| Body mass index, kg/m² | 21.9 ± 3.2 | 21.3 ± 3.5 | 0.115 |
| Pneumonia-triggered exacerbation | 248 (26.8%) | 29 (25.0%) | 0.673 |
| Heart rate before NIV, beats/min | 109.0 ± 11.4 | 110.3 ± 10.8 | 0.231 |
| Respiratory rate before NIV, breaths/min | 30.6 ± 4.8 | 31.3 ± 4.6 | 0.142 |
| Arterial pH before NIV | 7.276 ± 0.033 | 7.268 ± 0.033 | 0.021 |
| PaCO₂ before NIV, mmHg | 74.0 ± 9.9 | 75.6 ± 9.2 | 0.070 |
| NIV failure | 187 (20.2%) | 32 (27.6%) | 0.067 |

The displayed P values are descriptive. The comparison does not remove the possibility of selection bias because excluded records could not be fully reconciled at the source-record level.

**Supplementary Table S9. Complete dynamic-model equation and predictor coding.**

| **Symbol** | **Predictor** | **Input coding or unit** | **Coefficient (β)** |
| --- | --- | --- | --- |
| Intercept | Intercept | Not applicable | 12.35688836921864 |
| X1 | At least 2 AECOPD admissions in the previous year | 0 = no; 1 = yes | -0.15381148257578142 |
| X2 | Home oxygen therapy | 0 = no; 1 = yes | 0.01616711526394750 |
| X3 | Congestive heart failure | 0 = no; 1 = yes | 0.04359331569808727 |
| X4 | Pneumonia-triggered exacerbation | 0 = no; 1 = yes | 0.37334970444417720 |
| X5 | Heart rate before NIV | beats/min | 0.01383282661742241 |
| X6 | Respiratory rate before NIV | breaths/min | 0.05156381529469468 |
| X7 | Arterial pH before NIV | measured pH × 100 | -0.00086438967853842 |
| X8 | PaCO₂ before NIV | mmHg | 0.00834851049970974 |
| X9 | C-reactive protein | mg/L | -0.01159718433064338 |
| X10 | Albumin | g/L | 0.00117586583938970 |
| X11 | Time from hospital arrival to NIV | hours | -0.04030723713954652 |
| X12 | Respiratory rate at 2 hours | breaths/min | 0.05444567687719921 |
| X13 | Glasgow Coma Scale at 2 hours | points | -0.74284078066923680 |
| X14 | Arterial pH at 2 hours | measured pH × 100 | -0.00941301761420632 |
| X15 | PaO₂/FiO₂ at 2 hours | measured ratio ÷ 10 | -0.05494991178066348 |

Predicted probability is p = 1/[1 + exp(-LP)]. Binary predictors are coded 0 = no and 1 = yes. For pH variables, the measured value is multiplied by 100; PaO₂/FiO₂ at 2 hours is divided by 10. The accompanying offline calculator uses the same coefficients as the equation.

Complete linear predictor: LP = 12.356888 - 0.153811X1 + 0.016167X2 + 0.043593X3 + 0.373350X4 + 0.013833X5 + 0.051564X6 - 0.000864X7 + 0.008349X8 - 0.011597X9 + 0.001176X10 - 0.040307X11 + 0.054446X12 - 0.742841X13 - 0.009413X14 - 0.054950X15.

**Supplementary Table S10A. Components observed among the 187 NIV failures.**

| **Outcome component** | **n** | **Percentage of NIV failures** |
| --- | --- | --- |
| Endotracheal intubation | 152 | 81.3% |
| ICU transfer | 131 | 70.1% |
| In-hospital death | 22 | 11.8% |
| Failure within 24 h | 171 | 91.4% |

Components are not mutually exclusive. ICU, intensive care unit; NIV, noninvasive ventilation.

**Supplementary Table S10B. Mutually exclusive component patterns among the 187 NIV failures.**

| **Outcome pattern** | **n** | **Percentage of NIV failures** |
| --- | --- | --- |
| In-hospital death | 1 | 0.5% |
| ICU transfer | 32 | 17.1% |
| ICU transfer + In-hospital death | 2 | 1.1% |
| Intubation | 50 | 26.7% |
| Intubation + In-hospital death | 5 | 2.7% |
| Intubation + ICU transfer | 83 | 44.4% |
| Intubation + ICU transfer + In-hospital death | 14 | 7.5% |

Each patient is represented in one row pattern.

**Supplementary Table S10C. ICU-transfer-only events overall and by calendar-time cohort.**

| **Cohort** | **Composite events** | **ICU-transfer-only events** | **Percentage of composite events** |
| --- | --- | --- | --- |
| Overall analysis cohort | 187 | 32 | 17.1% |
| Development | 136 | 24 | 17.6% |
| Temporal validation | 51 | 8 | 15.7% |

The available records did not reliably establish whether NIV was continued and ultimately successful after ICU transfer. ICU-transfer-only events may therefore include escalation of monitoring location without definitive failure of the NIV modality.

**Supplementary Table S11. Assessment of nonlinearity for continuous and ordered predictors.**

| **Predictor** | **Functional-form assessment** | **Overall association P value** | **Nonlinearity P value** | **Nonlinear degrees of freedom** | **Resulting specification** |
| --- | --- | --- | --- | --- | --- |
| Heart rate before NIV | Natural cubic spline; knot locations: 90.00, 104.00, 113.15, 128.00 | 0.386 | 0.518 | 2 | Retain linear specification |
| Respiratory rate before NIV | Natural cubic spline; knot locations: 23.00, 29.00, 32.00, 39.00 | 0.053 | 0.166 | 2 | Retain linear specification |
| Arterial pH before NIV | Natural cubic spline; knot locations: 7.221, 7.261, 7.290, 7.328 | 0.976 | 0.900 | 2 | Retain linear specification |
| PaCO2 before NIV | Natural cubic spline; knot locations: 56.91, 70.08, 77.61, 90.19 | 0.684 | 0.593 | 2 | Retain linear specification |
| C-reactive protein | Natural cubic spline; knot locations: 9.15, 19.50, 29.30, 62.43 | 0.149 | 0.333 | 2 | Retain linear specification |
| Albumin | Natural cubic spline; knot locations: 30.30, 34.20, 36.70, 40.54 | 0.819 | 0.630 | 2 | Retain linear specification |
| Time from arrival to NIV | Natural cubic spline; knot locations: 0.70, 2.50, 4.60, 9.44 | 0.587 | 0.628 | 2 | Retain linear specification |
| Respiratory rate at 2 hours | Natural cubic spline; knot locations: 19.00, 27.00, 33.00, 41.00 | 0.050 | 0.209 | 2 | Retain linear specification |
| Glasgow Coma Scale at 2 hours | Ordinal-factor comparison; observed levels: 11, 12, 13, 14, 15 | <0.001 | 0.618 | 3 | Retain linear specification |
| Arterial pH at 2 hours | Natural cubic spline; knot locations: 7.232, 7.280, 7.306, 7.352 | 0.672 | 0.474 | 2 | Retain linear specification |
| PaO2/FiO2 at 2 hours | Natural cubic spline; knot locations: 110.9, 181.9, 220.0, 283.4 | 0.358 | 0.962 | 2 | Retain linear specification |

Each predictor was assessed separately in the fully adjusted development model. Natural cubic splines used four knots at the displayed empirical values. For 2-hour GCS, the linear trend was compared with an ordered categorical representation. P values assess evidence against the prespecified linear functional form.

**Supplementary Table S12A. Diagnostics for inverse-probability-of-inclusion weights.**

| **Cohort** | **N** | **Mean weight** | **SD weight** | **Minimum weight** | **1st percentile** | **Median** | **99th percentile** | **Maximum weight** | **Effective sample size** |
| --- | --- | --- | --- | --- | --- | --- | --- | --- | --- |
| Development | 692 | 1.000 | 0.043 | 0.926 | 0.932 | 0.993 | 1.119 | 1.128 | 690.700 |
| Temporal validation | 232 | 0.999 | 0.042 | 0.926 | 0.926 | 0.993 | 1.128 | 1.128 | 231.597 |

Stabilized weights were calculated within calendar-time cohort and truncated at the 1st and 99th percentiles. Effective sample size was calculated as (Σw)²/Σw².

**Supplementary Table S12B. Performance of the inverse-probability-of-inclusion-weighted dynamic model.**

| **Cohort** | **N** | **Events** | **AUC** | **AUC 95% CI** | **Calibration intercept** | **Calibration slope** | **Brier score** |
| --- | --- | --- | --- | --- | --- | --- | --- |
| Development | 692 | 136 | 0.806 | 0.767 to 0.845 | 0.000 | 1.000 | 0.122 |
| Temporal validation | 232 | 51 | 0.803 | 0.741 to 0.864 | 0.045 | 0.951 | 0.137 |

The weighted dynamic model was fitted in development and applied without refitting in temporal validation. AUC confidence intervals were estimated by 1,000 bootstrap resamples. Development calibration values are apparent in-sample estimates.

**Supplementary Table S12C. Comparison of primary and inverse-probability-of-inclusion-weighted coefficients.**

| **Predictor** | **Primary beta** | **IP-weighted beta** | **IP-weighted beta 95% CI** | **Beta difference** |
| --- | --- | --- | --- | --- |
| Intercept | 12.3569 | 12.6480 | -69.3942 to 94.6903 | 0.2912 |
| At least 2 AECOPD admissions in the previous year | -0.1538 | -0.1623 | -0.6093 to 0.2848 | -0.0085 |
| Home oxygen therapy | 0.0162 | 0.0074 | -0.5052 to 0.5200 | -0.0088 |
| Congestive heart failure | 0.0436 | 0.0529 | -0.5803 to 0.6861 | 0.0093 |
| Pneumonia-triggered exacerbation | 0.3733 | 0.3567 | -0.1108 to 0.8242 | -0.0166 |
| Heart rate before NIV | 0.0138 | 0.0139 | -0.0066 to 0.0343 | 0.0000 |
| Respiratory rate before NIV | 0.0516 | 0.0523 | -0.0012 to 0.1058 | 0.0007 |
| Arterial pH before NIV | -0.0009 | -0.0018 | -0.0732 to 0.0697 | -0.0009 |
| PaCO2 before NIV | 0.0083 | 0.0080 | -0.0155 to 0.0314 | -0.0004 |
| C-reactive protein | -0.0116 | -0.0110 | -0.0246 to 0.0026 | 0.0006 |
| Albumin | 0.0012 | -0.0024 | -0.0707 to 0.0659 | -0.0036 |
| Time from arrival to NIV | -0.0403 | -0.0390 | -0.1144 to 0.0364 | 0.0013 |
| Respiratory rate at 2 hours | 0.0544 | 0.0562 | 0.0023 to 0.1101 | 0.0018 |
| Glasgow Coma Scale at 2 hours | -0.7428 | -0.7378 | -1.0630 to -0.4127 | 0.0050 |
| Arterial pH at 2 hours | -0.0094 | -0.0089 | -0.0987 to 0.0808 | 0.0005 |
| PaO2/FiO2 at 2 hours | -0.0549 | -0.0530 | -0.1121 to 0.0061 | 0.0020 |

Beta difference is the weighted coefficient minus the primary unweighted coefficient. Weighting addresses selection associated with observed variables and does not remove unmeasured selection bias.

**Supplementary Table S13. Threshold sensitivity and exploratory comparator classification.**

**Panel A. Development cohort (N = 692; events = 136)**

| **Threshold type** | **Threshold** | **High-risk patients, n (%)** | **Failures among high-risk patients, n** | **Sensitivity** | **Specificity** | **PPV** | **NPV** | **Accuracy** | **Net benefit** |
| --- | --- | --- | --- | --- | --- | --- | --- | --- | --- |
| Fixed probability | 0.100 | 396 (57.2%) | 121 | 89.0% | 50.5% | 30.6% | 94.9% | 58.1% | 0.131 |
| Fixed probability | 0.150 | 313 (45.2%) | 110 | 80.9% | 63.5% | 35.1% | 93.1% | 66.9% | 0.107 |
| Fixed probability | 0.200 | 245 (35.4%) | 98 | 72.1% | 73.6% | 40.0% | 91.5% | 73.3% | 0.089 |
| Fixed probability | 0.250 | 189 (27.3%) | 84 | 61.8% | 81.1% | 44.4% | 89.7% | 77.3% | 0.071 |
| Fixed probability | 0.300 | 153 (22.1%) | 72 | 52.9% | 85.4% | 47.1% | 88.1% | 79.0% | 0.054 |
| Fixed probability | 0.400 | 97 (14.0%) | 55 | 40.4% | 92.4% | 56.7% | 86.4% | 82.2% | 0.039 |
| Fixed probability | 0.500 | 66 (9.5%) | 43 | 31.6% | 95.9% | 65.2% | 85.1% | 83.2% | 0.029 |
| Development-derived Youden (exploratory) | 0.166 | 290 (41.9%) | 109 | 80.1% | 67.4% | 37.6% | 93.3% | 69.9% | 0.106 |

**Panel B. Temporal validation cohort (N = 232; events = 51)**

| **Threshold type** | **Threshold** | **High-risk patients, n (%)** | **Failures among high-risk patients, n** | **Sensitivity** | **Specificity** | **PPV** | **NPV** | **Accuracy** | **Net benefit** |
| --- | --- | --- | --- | --- | --- | --- | --- | --- | --- |
| Fixed probability | 0.100 | 141 (60.8%) | 47 | 92.2% | 48.1% | 33.3% | 95.6% | 57.8% | 0.158 |
| Fixed probability | 0.150 | 115 (49.6%) | 43 | 84.3% | 60.2% | 37.4% | 93.2% | 65.5% | 0.131 |
| Fixed probability | 0.200 | 95 (40.9%) | 38 | 74.5% | 68.5% | 40.0% | 90.5% | 69.8% | 0.102 |
| Fixed probability | 0.250 | 78 (33.6%) | 34 | 66.7% | 75.7% | 43.6% | 89.0% | 73.7% | 0.083 |
| Fixed probability | 0.300 | 60 (25.9%) | 28 | 54.9% | 82.3% | 46.7% | 86.6% | 76.3% | 0.062 |
| Fixed probability | 0.400 | 36 (15.5%) | 20 | 39.2% | 91.2% | 55.6% | 84.2% | 79.7% | 0.040 |
| Fixed probability | 0.500 | 24 (10.3%) | 16 | 31.4% | 95.6% | 66.7% | 83.2% | 81.5% | 0.034 |
| Development-derived Youden (exploratory) | 0.166 | 109 (47.0%) | 42 | 82.4% | 63.0% | 38.5% | 92.7% | 67.2% | 0.124 |

**Panel C. Comparator classification at exploratory operating points**

| **Model** | **Cohort** | **Cutoff** | **Sensitivity** | **Specificity** | **Accuracy** | **PPV** | **NPV** |
| --- | --- | --- | --- | --- | --- | --- | --- |
| Pre-NIV model | Development | 0.272 | 52.2% | 82.7% | 76.7% | 42.5% | 87.6% |
| Pre-NIV model | Temporal validation | 0.272 | 49.0% | 85.1% | 77.2% | 48.1% | 85.6% |
| Dynamic model | Development | 0.166 | 80.1% | 67.4% | 69.9% | 37.6% | 93.3% |
| Dynamic model | Temporal validation | 0.166 | 82.4% | 63.0% | 67.2% | 38.5% | 92.7% |
| HACOR raw score | Development | 4.000 | 95.6% | 35.8% | 47.5% | 26.7% | 97.1% |
| HACOR raw score | Temporal validation | 4.000 | 98.0% | 33.1% | 47.4% | 29.2% | 98.4% |

Fixed probability thresholds and the development-derived Youden point are descriptive operating points and have not been validated as treatment thresholds. Panel C reports the development-derived Youden points for the pre-NIV and dynamic models and the conventional raw HACOR threshold of at least 4. Net benefit is reported for risk-guided reassessment at each probability threshold. NPV, negative predictive value; PPV, positive predictive value.

**Supplementary Table S14A. Performance of the full dynamic model and exploratory reduced models.**

**Panel A. Discrimination and overall prediction error**

| **Model** | **Cohort** | **N** | **Events** | **AUC (95% CI)** | **Brier score** |
| --- | --- | --- | --- | --- | --- |
| Primary 15-predictor dynamic model | Development | 692 | 136 | 0.807 (0.767 to 0.847) | 0.121 |
| Primary 15-predictor dynamic model | Temporal validation | 232 | 51 | 0.804 (0.741 to 0.866) | 0.137 |
| Four-variable 2-hour physiological model | Development | 692 | 136 | 0.795 (0.754 to 0.837) | 0.125 |
| Four-variable 2-hour physiological model | Temporal validation | 232 | 51 | 0.776 (0.711 to 0.840) | 0.148 |
| Three-variable 2-hour model (RR, GCS, PaO2/FiO2) | Development | 692 | 136 | 0.795 (0.754 to 0.837) | 0.125 |
| Three-variable 2-hour model (RR, GCS, PaO2/FiO2) | Temporal validation | 232 | 51 | 0.773 (0.708 to 0.838) | 0.149 |
| Two-variable 2-hour model (GCS and RR) | Development | 692 | 136 | 0.791 (0.750 to 0.832) | 0.127 |
| Two-variable 2-hour model (GCS and RR) | Temporal validation | 232 | 51 | 0.756 (0.689 to 0.823) | 0.152 |

**Panel B. Calibration**

| **Model** | **Cohort** | **Calibration intercept (95% CI)** | **Calibration slope (95% CI)** |
| --- | --- | --- | --- |
| Primary 15-predictor dynamic model | Development | 0.000 (-0.214 to 0.214) | 1.000 (0.810 to 1.190) |
| Primary 15-predictor dynamic model | Temporal validation | 0.041 (-0.315 to 0.398) | 0.939 (0.630 to 1.249) |
| Four-variable 2-hour physiological model | Development | 0.000 (-0.211 to 0.211) | 1.000 (0.805 to 1.195) |
| Four-variable 2-hour physiological model | Temporal validation | 0.029 (-0.324 to 0.383) | 0.831 (0.537 to 1.126) |
| Three-variable 2-hour model (RR, GCS, PaO2/FiO2) | Development | 0.000 (-0.211 to 0.211) | 1.000 (0.805 to 1.195) |
| Three-variable 2-hour model (RR, GCS, PaO2/FiO2) | Temporal validation | 0.032 (-0.321 to 0.386) | 0.818 (0.526 to 1.109) |
| Two-variable 2-hour model (GCS and RR) | Development | 0.000 (-0.210 to 0.210) | 1.000 (0.802 to 1.198) |
| Two-variable 2-hour model (GCS and RR) | Temporal validation | 0.020 (-0.331 to 0.371) | 0.792 (0.498 to 1.086) |

Each model was fitted in development and applied unchanged in temporal validation. Development calibration intercepts of 0 and slopes of 1 are apparent in-sample values and are not independent evidence of good calibration.

**Supplementary Table S14B. Paired discrimination comparisons in temporal validation.**

| **Comparator** | **ΔAUC: full minus comparator** | **ΔAUC 95% CI** | **Paired DeLong P value** |
| --- | --- | --- | --- |
| Four-variable 2-hour physiological model | 0.028 | 0.007 to 0.053 | 0.013 |
| Three-variable 2-hour model (RR, GCS, PaO2/FiO2) | 0.031 | 0.009 to 0.055 | 0.007 |
| Two-variable 2-hour model (GCS and RR) | 0.047 | 0.021 to 0.078 | <0.001 |

P values are from paired DeLong tests. Confidence intervals for ΔAUC were estimated with 1,000 paired nonparametric bootstrap resamples. The reduced models were exploratory and were not used to replace the prespecified primary model.

**Supplementary Table S15A. Descriptive development-sample-size assessment using the Riley framework.**

| **Criterion** | **Minimum sample size** | **Approximate expected events** | **Target/implied shrinkage** | **Events per parameter** | **Criterion met** |
| --- | --- | --- | --- | --- | --- |
| Criterion 1: expected shrinkage of at least 0.90 | 618 | 122 | 0.900 | 8.10 | Yes |
| Criterion 2: difference between apparent and adjusted Nagelkerke R2 no greater than 0.05 | 421 | 83 | 0.861 | 5.52 | Yes |
| Criterion 3: precise estimation of the overall outcome proportion | 243 | 48 | 0.900 | 3.18 | Yes |
| Final required sample size: maximum across criteria | 618 | 122 | 0.900 | 8.10 | Yes |

The retrospective study included all eligible patients and did not use an a priori recruitment calculation. The calculation used the observed development outcome proportion and apparent Cox-Snell R² (0.194) and is therefore post hoc and descriptive. Approximate expected events were obtained by multiplying the minimum sample size by the observed event proportion. The final requirement is the maximum across the three Riley criteria.

**Supplementary Table S15B. Development and temporal-validation sample-size summary.**

| **Item** | **Value** |
| --- | --- |
| Available development sample size | 692 |
| Available development events | 136 |
| Candidate predictor parameters | 15 |
| Observed events per predictor parameter | 9.07 |
| Development outcome proportion | 0.197 |
| Apparent Cox-Snell R2 | 0.194 |
| Maximum Cox-Snell R2 | 0.629 |
| Apparent Nagelkerke R2 | 0.309 |
| Target expected shrinkage | 0.900 |
| Riley-framework final minimum sample size | 618 |
| Approximate expected events corresponding to the final minimum sample size | 122 |
| Difference between available and required sample size | 74 |
| Difference between available and approximate expected events | 14 |
| Empirical bootstrap-corrected calibration slope | 0.876 |
| Temporal-validation sample size | 232 |
| Temporal-validation events | 51 |

The development cohort exceeded the nominal post hoc Riley-framework minimum, but the bootstrap-corrected calibration slope of 0.876 indicated residual overfitting. The temporal-validation cohort contained 51 events; calibration estimates, particularly in the upper-risk range, should be interpreted as imprecise.

# Supplementary figures

Supplementary Figure S1. Bootstrap optimism distributions for the primary dynamic model. Panel A shows the distribution of AUC optimism and Panel B the distribution of calibration-slope optimism across 1,000 resamples. Solid vertical lines show mean optimism and dashed vertical lines show zero.
